# Prognosis-based definition of left ventricular remodeling after ST-elevation myocardial infarction

**DOI:** 10.1007/s00330-018-5875-3

**Published:** 2018-12-13

**Authors:** Martin Reindl, Sebastian Johannes Reinstadler, Christina Tiller, Hans-Josef Feistritzer, Markus Kofler, Alexandra Brix, Agnes Mayr, Gert Klug, Bernhard Metzler

**Affiliations:** 1 University Clinic of Internal Medicine III, Cardiology and Angiology, Medical University of Innsbruck, Anichstrasse 35, A-6020 Innsbruck, Austria; 2 University Clinic of Cardiac Surgery, Medical University of Innsbruck, Anichstrasse 35, A-6020 Innsbruck, Austria; 3 University Clinic of Radiology, Medical University of Innsbruck, Anichstrasse 35, A-6020 Innsbruck, Austria

**Keywords:** ST-elevation myocardial infarction, Magnetic resonance imaging, Prognosis

## Abstract

**Objectives:**

Cardiac magnetic resonance (CMR) is the gold-standard modality for the assessment of left ventricular (LV) remodeling in ST-elevation myocardial infarction (STEMI) patients. However, the commonly used remodeling criteria have never been validated for hard clinical events. We therefore aimed to define clear CMR criteria of LV remodeling following STEMI with proven prognostic impact.

**Methods:**

This observational study included 224 patients suffering from acute STEMI. CMR was performed within 1 week and 4 months after infarction to evaluate different remodeling criteria including relative changes in LV end-diastolic volume (%∆LVEDV), end-systolic volume (%∆LVESV), ejection fraction (%∆LVEF), and myocardial mass (%∆LVMM). Primary endpoint was the occurrence of major adverse cardiovascular events (MACE) including all-cause death, re-infarction, stroke, and new congestive heart failure 24 months following STEMI. Secondary endpoint was defined as composite of primary endpoint and cardiovascular hospitalization. The Mann–Whitney *U* test was applied to assess differences in LV remodeling measures between patients with and without MACE. Values for the prediction of primary and secondary endpoints were assessed by *c*-statistics and Cox regression analysis.

**Results:**

The incidence of MACE (*n* = 13, 6%) was associated with higher %∆LVEDV (*p* = 0.002) and %∆LVMM (*p* = 0.02), whereas %∆LVESV and %∆LVEF were not significantly related to MACE (*p* > 0.05). The area under the curve (AUC) for the prediction of MACE was 0.76 (95% confidence interval [CI], 0.65–0.87) for %∆LVEDV (optimal cut-off 10%) and 0.69 (95%CI, 0.52–0.85) for %∆LVMM (optimal cut-off 5%). From all remodeling criteria, %∆LVEDV ≥ 10% showed highest hazard ratio (8.68 [95%CI, 2.39–31.56]; *p* = 0.001) for MACE. Regarding secondary endpoint (*n* = 35, 16%), also %∆LVEDV with an optimal threshold of 10% emerged as strongest prognosticator (AUC 0.66; 95%CI, 0.56–0.75; *p* = 0.004).

**Conclusions:**

Following revascularized STEMI, %∆LVEDV ≥ 10% showed strongest association with clinical outcome, suggesting this criterion as preferred CMR-based definition of post-STEMI LV remodeling.

**Key Points:**

• *CMR-determined %∆LVEDV and %∆LVMM were significantly associated with MACE following STEMI.*

• *Neither %∆LVESV nor %∆LVEF showed a significant relation to MACE.*

• *%∆LVEDV ≥ 10 was revealed as LV remodeling definition with highest prognostic validity.*

## Introduction

Adverse left ventricular (LV) remodeling following ST-elevation myocardial infarction (STEMI) is a maladaptive response to cardiac injury characterized by complex structural and functional myocardial changes [1]. Those maladaptive alterations occur at different myocardial levels affecting genome expression, molecular, cellular, and interstitial functions leading to LV dilation, dysfunction, and consequently, adverse clinical outcome [1]. However, the accurate determination of LV remodeling is still challenging, and until now, a consensus definition on LV remodeling is lacking [2]. A plethora of different parameters and different thresholds for the definition of LV remodeling can be found in the current literature [2–6]. Relative changes in LV end-diastolic volume (%∆LVEDV) and end-systolic volume (%∆LVESV) are the most common parameters used to define post-infarction LV remodeling [2, 7]. For those parameters, several LV remodeling-defining cut-off values exist; however, these thresholds were established by echocardiographic studies, primarily conducted in the era before primary percutaneous coronary intervention (PPCI), without any correlation to hard clinical events [2, 8].

Due to the low spatial resolution as well as limited reproducibility of echocardiography, cardiac magnetic resonance (CMR) imaging has emerged as preferred modality to evaluate LV remodeling over the last years [9–13]. However, for the definition of LV remodeling in the previous CMR studies, the echocardiographic-derived, prognostically questionable criteria were used without any CMR-specific validation [2]. Accordingly, there is a need for the development of a CMR-specific, prognosis-based definition of LV remodeling following STEMI.

We therefore aimed to comprehensively evaluate the associations of CMR-determined %∆LVEDV, %∆LVESV, %∆LV ejection fraction (LVEF), and %∆LV myocardial mass (LVMM) from the baseline to 4 months after STEMI with the occurrence of major adverse cardiovascular events (MACE) and determine thresholds of these remodeling parameters with highest prognostic value, developing new prognosis-based, CMR-specific criteria for the definition of LV remodeling.

## Methods

### Study design and endpoint definitions

In this prospective observational study, 228 consecutive STEMI patients presenting at the coronary care unit of the Innsbruck University Hospital were initially included. After exclusion of patients who died (*n* = 2) or developed a myocardial re-infarction (*n* = 2) until 4-month follow-up, a final cohort of 224 STEMI patients was analyzed. Inclusion criteria were defined as follows: age > 18 years, first ever STEMI according to the redefined ESC/ACC committee criteria [14] and revascularization by PPCI within 24 h after symptom onset. Exclusion criteria were an estimated glomerular filtration rate (eGFR) ≤ 30 ml/min/1.73 m^2^, Killip class ≥ 3, any absolute contraindication to CMR examination (pacemaker, orbital foreign body, cerebral aneurysm clip, and known contrast agent allergy to gadolinium), and death or myocardial re-infarction until 4-month follow-up investigation.

After inclusion, we conducted detailed medical history assessments as well as physical examinations and performed CMR scans within the first week after infarction. A follow-up CMR investigation was conducted at 4 months after the index event. This follow-up time point is based on previous CMR data showing that the first 4 months after STEMI represent the most dynamic phase in terms of LV functional changes [15].

Final assessment of clinical endpoints was conducted at 24 months after STEMI via telephone interview using a standardized questionnaire [16]. Patients were interviewed only by trained personnel blinded to CMR, angiographic, or clinical findings. The declared endpoints were carefully checked afterwards by reviewing the corresponding medical records.

Primary study endpoint was the development of MACE, defined as a composite of all-cause death, non-fatal myocardial re-infarction, stroke, or new congestive heart failure until 24-month follow-up. Re-infarction was defined in accordance with the redefined ESC/ACC committee criteria [14]. Stroke was defined according to the updated criteria of the American Stroke Association as ischemic or hemorrhagic stroke with an episode of neurological dysfunction due to focal cerebral infarction [17]. First episode of cardiac decompensation requiring diuretic therapy was used for the definition of new congestive heart failure [18]. Secondary endpoint was defined as composite of all-cause death, re-infarction, stroke, new congestive heart failure, and acute cardiovascular hospitalization within 24 months following STEMI.

Written informed consent was signed by each participant before inclusion. The study was conducted in accordance with the Declaration of Helsinki approved by the local research ethics committee.

### Cardiac magnetic resonance imaging

CMR scans were performed on a 1.5 Tesla Magnetom AVANTO-scanner (Siemens®). The applied imaging protocol was published in detail previously [19]. Briefly, short-axis (10 to 12 slices) cine images using breath-hold, retrospective ECG-triggered trueFISP bright-blood sequences were acquired to analyze LV morphology and function. Standard software (ARGUS, Siemens®) was used for post-processing [20]. LV volumes were evaluated semi-automatically by contouring the endo- and epicardial borders at end-diastolic and end-systolic images [10]. Papillary muscles were excluded from the myocardium and included into the LV volume.

At baseline CMR scans, also late gadolinium-enhanced (LGE) sequences were acquired to assess infarct size (IS) and particularly microvascular obstruction (MVO). For LGE imaging, an ECG-triggered phase-sensitive inversion recovery (PSIR) sequence was used. A PACS workstation (IMPAX, Agfa HealthCare®) was applied to determine the extent of “hyperenhancement,” defined as threshold of + 5 standard deviations above the signal intensity of remote myocardium. IS was presented as percentage of LVMM [21]. MVO was defined as “hypoenhancement” within the hyperenhanced infarct area [22].

### Statistical analysis

All statistical calculations were performed using SPSS Statistics 24.0 (IBM Corp.®), MedCalc Version 15.8 (MedCalc Software®), and R 3.3.0 (The R Foundation®). Continuous variables were expressed as median with interquartile range (IQR), and categorical variables were presented as number with corresponding percentage. Differences in continuous variables between two groups were evaluated with the Mann–Whitney *U* test. The chi-square test was used to assess differences in categorical variables between groups. Correlations between LV remodeling parameters were evaluated by using Spearman’s rank test. Receiver operating characteristic (ROC) curve analysis was applied to determine the area under the curve (AUC) for the prediction of MACE; the optimal cut-off values were determined via the Youden index [23]. Univariable and multivariable Cox regression models were calculated to identify significant correlates of MACE. The remodeling cut-offs applied in univariable analysis were either the ROC-determined best cut-offs or the thresholds commonly used in the literature (%∆LVEDV 20% or 12% [20, 24, 25]; %∆LVESV 15% or 12% [25, 26]). In consideration of the moderate MACE rate, only two variables (%∆LVEDV ≥ 10% and one further variable for each model) were included into multivariable regression to warrant statistical reliability. MACE-free survival was estimated and illustrated by means of the Kaplan–Meier method while differences in MACE-free survival were evaluated using the log-rank test. To evaluate additive prognostic impact of %∆LVMM over %∆LVEDV, net reclassification improvement (NRI) and integrated discrimination improvement (IDI) were calculated by using R package “PredictABEL.” Cut-off values for risk classes were defined according to the MACE rates obtained by forming groups on the basis of LV remodeling determined by %∆LVEDV or/and %∆LVMM (1.6%, 5.1%, 31.8%). To emphasize the reclassification improvement independently of risk categories, the continuous NRI was also calculated. For all analyses, a two-tailed *p* value of < 0.05 was defined as statistically significant.

## Results

### Baseline characteristics

Two hundred twenty-four STEMI patients with a median ischemia time of 206 (IQR, 135 to 389) min were enrolled. All baseline characteristics are listed in Table 1. Patients received state of the art contemporary post-STEMI treatment: dual antiplatelet therapy (*n* = 223, 99%), beta-blocker (*n* = 196, 88%), angiotensin-converting-enzyme inhibitor or angiotensin receptor blocker (*n* = 200, 89%), and statin (*n* = 220, 98%).

**Table 1:** Patient characteristics

Characteristic	Total population (*n* = 224)	No MACE (*n* = 211, 94%)	MACE (*n* = 13, 6%)	*p* value
Age, years	56 (49–66)	56 (49–66)	61 (49–73)	0.35
Female, *n* (%)	33 (15)	30 (14)	3 (23)	0.38
Body mass index (kg/m^2^)	26.2 (24.7–28.4)	26.2 (24.7–28.6)	25.8 (23.3–27.2)	0.17
Hypertension, *n* (%)	129 (58)	120 (57)	9 (69)	0.39
Systolic blood pressure (mmHg)	127 (114–147)	127 (113–146)	135 (121–150)	0.55
Diastolic blood pressure (mmHg)	80 (70–90)	80 (70–90)	83 (75–95)	0.35
Family history for AMI, *n* (%)	59 (26)	56 (27)	3 (23)	0.93
Current smoker, *n* (%)	127 (57)	118 (56)	9 (69)	0.36
Hyperlipidemia, *n* (%)	142 (63)	134 (64)	8 (62)	0.87
Diabetes mellitus, *n* (%)	23 (10)	18 (9)	5 (39)	*0.001*
Culprit lesion				0.36
RCA	99 (44)	94 (45)	5 (38)	
LAD	94 (42)	86 (41)	8 (62)	
LCX	28 (13)	28 (13)	0 (0)	
RI	3 (1)	3 (1)	0 (0)	
Time from symptom onset to PPCI (min)	206 (135–389)	206 (136–389)	177 (103–494)	0.38
Pre-interventional TIMI flow				0.49
0	135 (60)	126 (60)	9 (69)	
1	34 (15)	31 (15)	3 (23)	
2	45 (20)	44 (21)	1 (8)	
3	10 (5)	10 (5)	0 (0)	
Post-interventional TIMI flow				*0.002*
0	7 (3)	6 (3)	1 (8)	
1	3 (1)	2 (1)	1 (8)	
2	19 (9)	15 (7)	4 (31)	
3	195 (87)	188 (89)	7 (54)	

All continuous variables are presented as median (interquartile range), categorical variables as number (percentage). All *p* values refer to differences in continuous variables (Mann–Whitney *U* test) or categorical variables (chi-square test) between patients with and without MACE. *P*-values < 0.05 are highlighted in italics

*MACE* major adverse cardiovascular event, *AMI* acute myocardial infarction, *RCA* right coronary artery, *LAD* left anterior descending artery, *LCX* left circumflex artery, *RI* ramus intermedius, *PPCI* primary percutaneous coronary intervention

### Baseline and follow-up CMR findings

CMR scans were conducted 2 (IQR, 2 to 4) days as well as 128 (IQR, 122 to 136) days following PPCI. The baseline and follow-up CMR parameters as well as their relative changes are provided in detail by Table 2 (column “Total population”). Body surface area (BSA) did not relevantly change within 4 months (baseline BSA 2.0 [IQR 1.8 to 2.1] m^2^, follow-up BSA 2.0 [IQR 1.8 to 2.1] m^2^, absolute difference 0.00 [IQR 0.00–0.03] m^2^); therefore, relative changes in CMR parameters were only calculated for raw volumes and mass, not for indexed data. %∆LVEDV was significantly correlated to %∆LVESV (*r* = 0.69, *p* < 0.001) and %∆LVMM (*r* = 0.22, *p* = 0.001) but not to %∆LVEF (*r* = -0.08, *p* = 0.26). %∆LVESV showed a significant association with %∆LVEF (*r* = 0.68, *p* < 0.001), but not with %∆LVMM (*r* = 0.04, *p* = 0.54). The correlation between %∆LVEF and %∆LVMM was weak but significant (*r* = 0.15, *p* = 0.03).

**Table 2:** CMR parameters

CMR parameter	Total population (*n* = 224)	No MACE (*n* = 211, 94%)	MACE (*n* = 13, 6%)	*p* value
LVEF baseline (%)	54 (47-60)	54 (47–60)	42 (37-55)	*0.02*
LVEDV baseline (ml)	151 (125-167)	151 (127-166)	128 (102-171)	0.32
LVEDVi baseline (ml/m^2^)	74.7 (66.7-82.6)	74.8 (67.4-82.5)	66.7 (58.1-85.3)	0.42
LVESV baseline (ml)	69 (51-83)	68 (52-83)	79 (46-109)	0.39
LVESVi baseline (ml/m^2^)	34.9 (27.3-42.0)	34.8 (27.3-41.4)	47.6 (27.1-52.7)	0.23
LVMM baseline (g)	134 (115-156)	135 (116-155)	132 (94-154)	0.38
LVMMi baseline (g/m^2^)	67.9 (60.1-76.8)	68.0 (60.4-77.0)	66.0 (55.7-76.5)	0.34
CO baseline (ml/min)	5.4 (4.6-6.2)	5.4 (4.6-6.2)	4.8 (4.1-5.8)	0.09
CI baseline (ml/min/m^2^)	2.8 (2.4-3.1)	2.7 (2.4-3.1)	2.7 (2.1-2.9)	0.16
IS baseline (% of LVMM)	15 (7-26)	15 (7-25)	21 (15-41)	0.12
MVO, *n* (%)	118 (53)	107 (51)	11 (85)	*0.01*
LVEF follow-up (%)	59 (51-65)	59 (51-65)	51 (40-61)	*0.04*
LVEDV follow-up (ml)	149 (127-168)	148 (127-167)	153 (117-194)	0.64
LVEDVi follow-up (ml/m^2^)	75.0 (65.4-85.9)	74.8 (65.2-85.5)	82.6 (65.9-98.4)	0.20
LVESV follow-up (ml)	60 (49-79)	60 (49-78)	89 (44-107)	0.14
LVESVi follow-up (ml/m^2^)	31.3 (23.9-39.6)	31.3 (23.9-38.6)	41.6 (23.6-58.7)	0.07
LVMM follow-up (g)	128 (114-148)	128 (114-48)	127 (99-159)	0.99
LVMMi follow-up (g/m^2^)	68.9 (58.3-73.4)	64.7 (58.3-72.8)	69.1 (58.2-78.7)	0.52
CO follow-up (ml/min)	5.3 (4.5-5.9)	5.3 (4.5-5.9)	4.8 (4.4-6.7)	0.72
CI follow-up (ml/min/m^2^)	2.7 (2.3-3.0)	2.7 (2.3-3.0)	2.6 (2.4-3.2)	0.88
%∆LVEF (%)	8 (-1–18)	8 (-1–18)	9 (-6–32)	0.66
%∆LVEDV (%)	2 (- 9-12)	1 (- 9-10)	13 (5-25)	*0.002*
%∆LVESV (%)	- 6 (- 21-9)	-6 (-21–9)	6 (-9–23)	0.06
%∆LVMM (%)	-4 (-14–5)	- 5 (- 14-4)	6 (-7–22)	*0.02*
%∆CO (%)	-4 (-15–10)	-4 (-14–5)	4 (-11–29)	0.11

All continuous variables are presented as median (interquartile range), categorical variables as number (percentage). All *p* values refer to differences in continuous variables (Mann–Whitney *U* test) or categorical variables (chi-square test) between patients with and without MACE. *P*-values < 0.05 are highlighted in italics

*CMR* cardiac magnetic resonance, *MACE* major adverse cardiovascular events, *LVEF* left ventricular ejection fraction, *LVEDV* left ventricular end-diastolic volume, *LVEDVi* left ventricular end-diastolic volume index, *LVESV* left ventricular end-systolic volume, *LVESVi* left ventricular end-systolic volume index, *LVMM* left ventricular myocardial mass, *LVMMi* left ventricular myocardial mass index, *CO* cardiac output, *CI* cardiac index, *IS* infarct size, *MVO* microvascular obstruction

### Health outcome

All 224 patients could be followed for health outcome. Median follow-up time was 24 (IQR, 24 to 24) months. Thirteen patients (6%) developed a MACE event (2 patients died, 3 patients suffered from a myocardial re-infarction, 6 patients developed a new congestive heart failure, and 2 patients experienced a stroke). The baseline characteristics and CMR parameters according to the absence or presence of MACE are shown by Tables 1 and 2, respectively. Patients with MACE were more frequently diabetics (*p* = 0.001) and had a significantly worse post-interventional TIMI flow (*p* = 0.002). Concerning CMR parameters, MACE was significantly associated with baseline (*p* = 0.02) and follow-up LVEF (*p* = 0.04) as well as with MVO (*p* = 0.01).

The secondary clinical endpoint occurred in 35 patients (16%). Reasons for acute cardiovascular hospitalization (*n* = 22, 10%) were symptoms suggestive of acute myocardial ischemia (*n* = 20) and syncope (*n* = 2).

### Association between CMR parameters of remodeling and clinical outcome

**Fig. 1:**
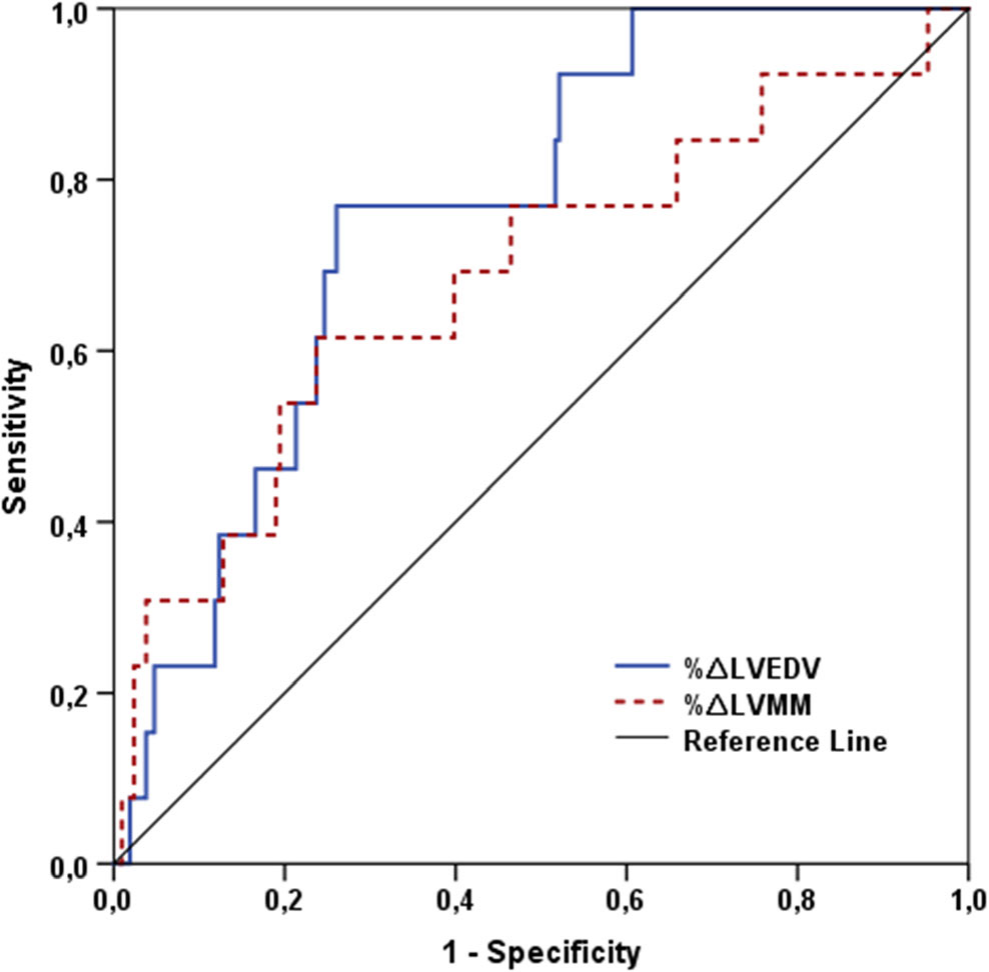
ROC curves of %∆LVEDV and %∆LVMM for the prediction of MACE. ROC, receiver operating characteristic; LVEDV, left ventricular end-diastolic volume; LVMM, left ventricular myocardial mass; MACE, major adverse cardiovascular events

From the CMR parameters indicating LV remodeling (%∆LVEDV, %∆LVESV, %∆LVEF, and %∆LVMM), only %∆LVEDV (*p* = 0.002) and %∆LVMM (*p* = 0.02) were significantly associated with MACE. In ROC analysis, the AUC for the prediction of MACE was 0.76 (95% confidence interval (CI) 0.65 to 0.87) for %∆LVEDV with an optimal cut-off of 10%, and 0.69 (95%CI 0.52 to 0.85) for %∆LVMM with 5% as optimal cut-off (Fig. 1). The AUC values of the remodeling parameters not significantly associated with MACE were as follows: %∆LVESV 0.65 (95%CI 0.52 to 0.80; best cut-off 6%) and %∆LVEF 0.46 (95%CI 0.27 to 0.66).

The results from the univariable and multivariable Cox regression analyses are shown by Tables 3 and 4, respectively. For prediction of MACE, %∆LVEDV ≥ 10% had the highest hazard ratio (8.68, 95%CI, 2.39 to 31.56). In total, 65 patients (29%) showed %∆LVEDV ≥ 10%. From these 65 patients, 10 developed a MACE event (15%). Patients with %∆LVEDV ≥ 10% to %∆LVEDV < 20% (*n* = 36) had a MACE rate of 17% (*n* = 6), whereas patients with %∆LVEDV ≥ 20% (*n* = 29) had a MACE rate of 14% (*n* = 4). Neither %∆LVEDV ≥ 20% nor %∆LVESV ≥ 15% was significantly associated with MACE (both *p* > 0.05; Table 3). The association between %∆LVEDV ≥ 10% and MACE also remained significant after adjustment for other significant determinants of MACE (Table 4). Also, %∆LVMM ≥ 5% remained significantly associated with MACE after adjusting for all those parameters (all *p* ≤ 0.02).

**Table 3:** Univariable Cox regression analysis for prediction of MACE

	HR (95%CI)	*p* value
%∆LVEDV (%)	1.04 (1.02–1.07)	*0.003*
%∆LVEDV ≥ 10%	8.68 (2.39–31.56)	*0.001*
%∆LVEDV ≥ 12%	4.11 (1.38–12.23)	*0.01*
%∆LVEDV ≥ 20%	3.17 (0.98–10.28)	0.06
%∆LVESV (%)	1.01 (0.99–1.03)	0.13
%∆LVESV ≥ 6%	3.02 (1.02–8.99)	*0.05*
%∆LVESV ≥ 12%	2.38 (0.78–7.28)	0.13
%∆LVESV ≥ 15%	2.84 (0.93–8.69)	0.07
%∆LVMM (%)	1.05 (1.01–1.08)	*0.01*
%∆LVMM ≥ 5%	4.79 (1.57–14.65)	*0.01*
LVEF baseline	0.94 (0.89–0.98)	*0.01*
LVEF baseline < 47%	4.67 (1.53–14.27)	*0.01*
LVEF follow-up	0.94 (0.90–0.98)	*0.01*
LVEF follow-up < 52%	4.23 (1.39–12.94)	*0.01*
MVO	9.88 (1.28–76.56)	*0.03*
Diabetes mellitus	5.92 (1.94–18.10)	*0.002*
Post-interventional TIMI flow	0.52 (0.32–0.84)	*0.01*

*P*-values < 0.05 are highlighted in italics

*MACE* major adverse cardiovascular events, *HR* hazard ratio, *LVEDV* left ventricular end-diastolic volume, *LVESV* left ventricular end-systolic volume, *LVMM* left ventricular myocardial mass, *LVEF* left ventricular ejection fraction, *MVO* microvascular obstruction

**Table 4:** Multivariable Cox regression analysis for prediction of MACE

	HR (95%CI)	*p* value
Model A		
%∆LVEDV ≥ 10%	8.59 (2.04–36.20)	*0.003*
%∆LVESV ≥ 6%	1.02 (0.30–3.44)	0.97
Model B		
%∆LVEDV ≥ 10%	7.53 (2.06–27.52)	*0.002*
%∆LVMM ≥ 5%	3.89 (1.27–11.97)	*0.02*
Model C		
%∆LVEDV ≥ 10%	7.22 (1.96–26.54)	*0.003*
LVEF baseline < 47%	3.52 (1.14–10.87)	*0.03*
Model D		
%∆LVEDV ≥ 10%	6.67 (1.75–25.35)	*0.005*
LVEF follow-up < 52%	2.50 (0.78–7.94)	0.12
Model E		
%∆LVEDV ≥ 10%	5.92 (1.59–22.06)	*0.01*
MVO	6.93 (0.88–54.44)	0.07
Model F		
%∆LVEDV ≥ 10%	8.39 (2.31–30.51)	*0.001*
Diabetes mellitus	5.64 (1.84–17.27)	*0.002*
Model G		
%∆LVEDV ≥ 10%	11.12 (2.92–42.41)	*< 0.001*
Post-interventional TIMI flow	0.41 (0.24–0.70)	*0.001*

Considering the moderate event rate, multivariable Cox regression analysis was restricted to a maximum of 2 variables (%∆LVEDV ≥ 10% and one further variable), resulting in seven regression models (Model A–G). *P*-values < 0.05 are highlighted in italics

*MACE* major adverse cardiovascular events, *HR* hazard ration, *LVEDV* left ventricular end-diastolic volume, *LVESV* left ventricular end-systolic volume, *LVMM* left ventricular myocardial mass, *LVEF* left ventricular ejection fraction

**Fig. 2:**
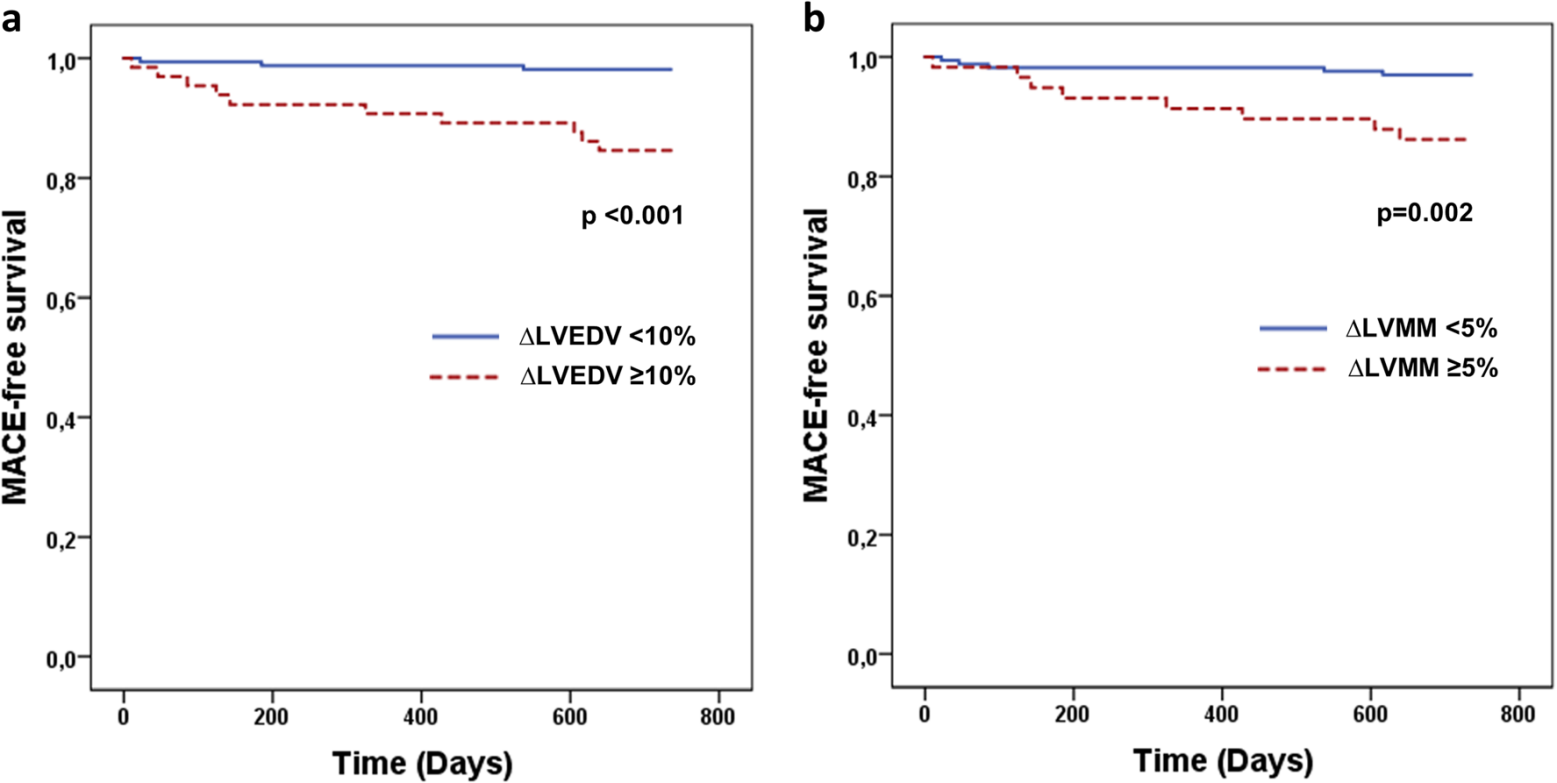
Kaplan–Meier curves displaying MACE-free survival according to the presence or absence of LV remodeling as defined by ∆LVEDV ≥ 10% (panel **a**) or ∆LVMM ≥ 5% (panel **b**). MACE, major adverse cardiovascular events; LVEDV, left ventricular end-diastolic volume; LVMM, left ventricular myocardial mass

As illustrated by the Kaplan–Meier curves (Fig. 2), %∆LVEDV ≥ 10% (*p* < 0.001) and %∆LVMM ≥ 5% (*p* = 0.002) were associated with significantly lower MACE-free survival.

**Fig. 3:**
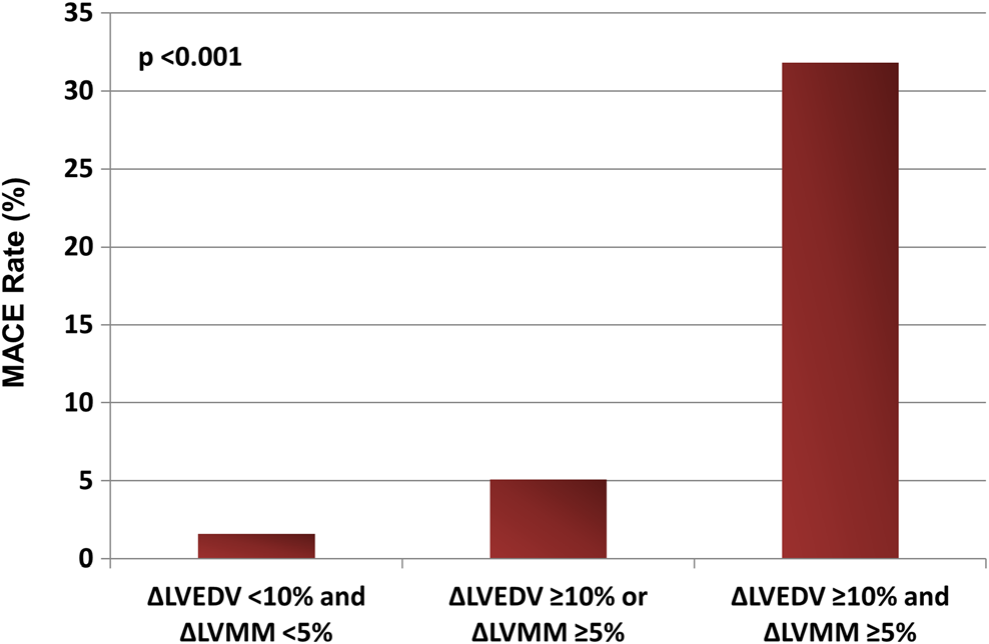
Relation of LV remodeling defined by ∆LVEDV ≥ 10% or/and ∆LVMM ≥ 5% with MACE. LVEDV, left ventricular end-diastolic volume; LVMM, left ventricular myocardial mass; MACE, major adverse cardiovascular events

The relation between LV remodeling defined by %∆LVEDV ≥ 10% or %∆LVMM ≥ 5% and MACE is shown by Fig. 3: Patients without remodeling (%∆LVEDV < 10% and %∆LVMM < 5%) had a MACE rate of 1.6% (*n* = 2 of 123), patients with either %∆LVEDV ≥ 10% or %∆LVMM ≥ 5% had a MACE rate of 5.1% (*n* = 4 of 79), and patients with both %∆LVEDV ≥ 10% and %∆LVMM ≥ 5% showed a MACE rate of 31.8% (*n* = 7 of 22; *p* < 0.001). The addition of %∆LVMM ≥ 5% to %∆LVEDV ≥ 10% led to a significant improvement in MACE prediction (NRI 0.42 [95%CI 0.21 to 0.63], *p* < 0.001; continuous NRI 0.76 [95%CI 0.22 to 1.30], *p* = 0.006; IDI 0.06 [95%CI 0.01 to 0.11], *p* = 0.02), and the AUC of the combined variable was 0.81 (95%CI 0.67 to 0.95).

For the prediction of the secondary clinical endpoint, the following AUC values were ascertained: %∆LVEDV 0.66 (95%CI 0.56 to 0.75, *p* = 0.004; optimal cut-off 10%), %∆LVESV 0.63 (95%CI 0.53 to 0.74, *p* = 0.01; optimal cut-off 3%), %∆LVMM 0.63 (95%CI 0.52 to 0.74, *p* = 0.01; optimal cut-off 4%), and %∆LVEF 0.45 (95%CI 0.34 to 0.56, *p* = 0.32).

## Discussion

This is the first CMR study aiming to define criteria for LV remodeling with strongest correlation to clinical complications in contemporary STEMI patients treated with PPCI. The key findings can be summarized as follows: (1) %∆LVEDV and %∆LVMM were significantly associated with MACE at 24 months after STEMI, whereas %∆LVESV or %∆LVEF did not show a significant relation with MACE. (2) %∆LVEDV provided highest prognostic value, and the optimal cut-off for the prediction of MACE was 10%; the optimal cut-off for %∆LVMM was 5%, and (3) the combination of %∆LVEDV ≥ 10% with %∆LVMM ≥ 5% provided incremental prognostic information.

### CMR definition of LV remodeling

The strong clinical impact of adverse LV remodeling following myocardial infarction has been recognized decades ago [27], and multiple studies conducted in the pre-PPCI and contemporary era have highlighted the association of LV remodeling with worse cardiovascular outcome [24, 28, 29]. However, the search for the best definition of post-infarction LV remodeling has been challenging [2].

Due to its broad availability, echocardiography has been primarily applied for the assessment of LV remodeling in both research and daily clinical routine [2]. The most frequent thresholds used to define LV remodeling were 20% increase in LVEDV and 15% increase in LVESV [2]. However, both cut-offs were not determined on the basis of clinical outcome but are rather the product of long citation flows ending with McKay et al [30] and Mollema et al [31], respectively [2].

Despite the well-known differences between CMR and echocardiography [9, 12], these echocardiographic thresholds were also applied in all previous CMR studies without any further validation [2]. Especially, the increase in LVEDV of 20% has been the most commonly used definition in CMR [2]. Eventually in 2017, Bulluck et al aimed to determine CMR-specific definitions of LV remodeling and proposed lower thresholds [25]. They suggested an increase of 12% for both LVEDV and LVESV as CMR definition of LV remodeling, based on the minimal detectable changes in those LV parameters from baseline to follow-up scan [25]. However, despite an overall available STEMI cohort of 146 patients, these minimal detectable changes are derived from a subgroup of only 40 patients [25]. Above and beyond this small number of patients, a major limitation of the study by Bulluck and colleagues is that the remodeling cut-offs have not been correlated with clinical events [25].

By the present study, we provide first-time data on the relation of CMR-determined LV parameters with hard clinical endpoints following revascularized STEMI. One of the most relevant findings of our investigation is that from all remodeling parameters, %∆LVEDV provided highest discrimination for adverse clinical outcome. Moreover, not the echocardiographic-derived and most frequently used cut-off of 20%, but 10% emerged as best ∆LVEDV threshold. Notably, ∆LVEDV ≥ 20% even failed to significantly predict MACE in univariable Cox regression analysis. To better understand the rationale behind this suggested cut-off reduction, it is crucial to consider the natural course of LVEDV within the first months after STEMI. The vast majority of STEMI patients develop no or only a minimal increase in %∆LVEDV [25]. Indeed, in the present cohort, 71% showed an increase in %∆LVEDV of < 10%, and nearly half of the overall population (concretely 45%) even showed a decrease in %∆LVEDV. Accordingly, patients with %∆LVEDV ≥ 10% in general represent a minority, and a further differentiation revealed that the subgroup with %∆LVEDV ≥ 10% but < 20% and the subgroup with %∆LVEDV ≥ 20% were comparable in size. More importantly, also the MACE rates in those subgroups were comparable (even slightly higher in the group showing %∆LVEDV ≥ from 10 to < 20%: 17% vs. 14%). The MACE rate on the other side of the %∆LVEDV spectrum (%∆LVEDV < 10%) was very low (3%). Therefore, the patient group with %∆LVEDV < 10% could be interpreted as low-risk group, whereas both other subgroups could be interpreted as high-risk groups with, importantly, similar risk of MACE, which clearly justifies and suggests a reduction of the %∆LVEDV cut-off to 10%. It is important to emphasize that this novel threshold of 10% not only emerged as best correlate of hard clinical events (MACE) but also showed highest association with “softer” clinical complications including cardiovascular rehospitalization (secondary endpoint), underscoring and corroborating the clinical significance of this new cut-off. This lowering of the %∆LVEDV cut-off for CMR analyses would be in full agreement with the data by Bulluck et al which already indicated that 20% might be an overestimated threshold for CMR [25], explainable by the higher spatial resolution as well as reproducibility of CMR as compared to echocardiography [25].

%∆LVESV provided less prognostic information than %∆LVEDV in our analyses, showing even a non-significant relation to MACE, and %∆LVEF was demonstrated to be an inadequate correlate of MACE. The poor prognostic value of %∆LVEF could be explained by the fact that despite the significantly lower baseline LVEF, patients with MACE underwent a quantitative LVEF recovery comparable with the patient group without MACE through amplified functional compensation as reflected by the hypertrophic remodeling pattern in MACE patients (higher %∆LVMM). In line with this concept, in MACE patients cardiac output (CO) tended to increase, whereas patients without MACE showed a slight decrease of %∆CO. These processes in turn could help explain the limited prognostic accuracy of %∆LVESV detected in our analysis. Since %∆LVESV basically emerges from both %∆LVEDV and %∆LVEF, the influence of %∆LVEF could markedly diminish the prognostic value of %∆LVESV.

### Combination of CMR remodeling parameters

Parameters of global LV volume and function have become the standard measures for the assessment of LV remodeling [2, 10]; however, it is largely unclear whether the combination of remodeling parameters is beneficial. In the recently published CMR study by Bulluck et al, they combined %∆LVEDV and %∆LVESV with the aim to better characterize different remodeling patterns [25]. However, these different remodeling groups derived, as already mentioned above, from cut-offs determined by minimal detectable CMR changes of a rather arbitrarily defined cohort of 40 patients without any correlation to clinical parameters [25]. Our prognosis-based data indicate that the combination of %∆LVEDV and %∆LVESV is not beneficial. As demonstrated by the logistic regression analysis, the relation between %∆LVESV and MACE completely declined when adjusting for %∆LVEDV. Accordingly, the prognostic value of %∆LVESV seems to primarily emerge from its %∆LVEDV component, reflected by the good correlations between those two remodeling parameters detected.

The processes of post-STEMI adverse remodeling are very complex and characterized not only by LV enlargement and dysfunction but also by simultaneous compensatory mechanisms [27]. One crucial mechanism in this compensatory cascade is myocyte hypertrophy leading to an increase in LVMM, which is referred to as hypertrophic remodeling [27]. In the present analysis, we could reveal that in patients developing MACE, this hypertrophic remodeling occurs to a high degree preserving LV functional recovery within the first months after STEMI. Importantly, the relation between %∆LVMM and clinical outcome was not only significant but also independent from %∆LVEDV. Moreover, the combination of %∆LVEDV with %∆LVMM even led to a significant improvement in risk classification, suggesting that the additional evaluation of %∆LVMM may be useful in clinical practice.

### Clinical data

Above and beyond remodeling measures per se, the present analysis could also highlight the prognostic relevance of different clinical parameters. First, manifest diabetes mellitus was shown to significantly predict the development of cardiovascular complications in the present cohort. This finding is, despite the moderate MACE rate as well as diabetes prevalence in the present population, in full agreement with the existing literature which proposes that diabetes is not only one of the major risk factors for the development of myocardial infarction [32] but also acts as crucial determinant of worse outcome after infarction [33, 34]. Accordingly, diabetes mellitus has emerged as crucial tool for risk stratification of STEMI patients in clinical routine, implemented in different risk scoring systems including the TIMI risk score and the recently established ACEF-STEMI score [34]. In multivariable analysis of the present cohort, diabetes was shown do predict MACE independently of %∆LVEDV ≥ 10%, suggesting that the evaluation of diabetes status additionally to remodeling may be beneficial in terms of prognosis assessment post-STEMI. These multivariable results, in turn, also indicate that diabetes did not relevantly confound the relation between %∆LVEDV ≥ 10% and the primary endpoint.

Post-interventional TIMI flow was detected as second univariable and multivariable clinical predictor of MACE, which is in line with previous large multicenter analyses [35]. Insufficient coronary flow after PPCI often is the result of obstructions and destructions of the myocardial microvasculature, a constellation which promotes processes of adverse LV remodeling and favors worse clinical outcome [35]. We evaluated dysfunctions of the microvascular by using CMR, the gold-standard modality for MVO assessment, and could once again emphasize the strong prognostic value of MVO. In multivariable testing including %∆LVEDV ≥ 10% and MVO, the significant association between MVO and MACE dissolved, revealing MVO as one important but not the only underlying pathomechanism in the context of LV remodeling and clinical outcome following STEMI.

### Limitations

We acknowledge that our study has limitations. Although this is the largest CMR study addressing the relation of LV remodeling criteria with clinical outcome following STEMI, the sample size and particularly event rate are still moderate. Hence, external validation of our findings by multicenter studies is necessary. Furthermore, only stable STEMI patients with Killip class < 3 were included; therefore, our data cannot be generalized to unstable STEMI patients presenting with manifest pulmonary edema or cardiogenic shock. Finally, the present study was designed to assess and compare the established volumetric parameters of LV remodeling; more recently emerged parameters of tissue characterization (e.g., T1 and T2 mapping [29]) associated with adverse remodeling have not been evaluated.

## Conclusions

In STEMI patients undergoing PPCI, %∆LVEDV with a cut-off value of 10% showed highest prognostic impact, proposing this threshold as preferred CMR definition of LV remodeling. Moreover, the evaluation of %∆LVMM additionally to %∆LVEDV may be helpful to further improve risk stratification of this patient population.

## Abbreviations

AUC: Area under the curve
CI: Confidence interval
CMR: Cardiac magnetic resonance
eGFR: Estimated glomerular filtration rate
IDI: Integrated discrimination improvement
IQR: Interquartile range
LV: Left ventricular
LVEDV: Left ventricular end-diastolic volume
LVEF: Left ventricular ejection fraction
LVESV: Left ventricular end-systolic volume
LVMM: Left ventricular myocardial mass
MACE: Major adverse cardiovascular events
MVO: Microvascular obstruction
NRI: Net reclassification improvement
PPCI: Primary percutaneous coronary intervention
ROC: Receiver operating characteristic
STEMI: ST-elevation myocardial infarction
